# Morphometric characterization of collagen and fat in normal ventricular myocardium

**DOI:** 10.1016/j.carpath.2020.107224

**Published:** 2020

**Authors:** Chris Miles, Joseph Westaby, Irina Chis Ster, Angeliki Asimaki, Peter Boardman, Adwait Joshi, Michael Papadakis, Sanjay Sharma, Elijah R. Behr, Mary N. Sheppard

**Affiliations:** a Cardiology Clinical Academic Group, Molecular and Clinical Sciences Institute, St George's, University of London, London, United Kingdom; b Institute of Infection and Immunity, St George's, University of London, London, United Kingdom

**Keywords:** Digital quantification, Myocardial fibrosis, Collagen, Fat, Normal heart

## Abstract

**Objective:**

We used automated image analysis software to determine the proportion of collagen, fat, and myocytes across six histological regions of normal ventricular myocardium.

**Methods:**

Twenty-nine non-cardiac death cases referred to our national cardiac pathology center were included in this study. Whole hearts were macroscopically and microscopically normal following expert histopathological evaluation. Tissue sections from the right ventricular outflow tract, right ventricle (RV), anterior interventricular septum (IVS), posterior IVS, anterior left ventricle (LV), and posterior LV were stained with Picrosirius red for collagen and scanned using a high-resolution slide scanner. Quantification of collagen, fat, and myocyte proportions was performed using Visiopharm software after automated exclusion of perivascular collagen.

**Results:**

The majority of decedents were male (25/29; 86%) with a mean age at death of 32.1 ± 9.9 (range 18-54) and mean BMI 28.7 ± 7.3. We report predicted values (collagen %, fat %, myocytes %) for cardiac tissue composition within the RV, IVS, and LV (including epicardial and endocardial layers). The proportion of collagen and fat were higher in the RV compared with the LV (ratios 1.61 [1.45-1.78]; 2.63 [1.99-3.48], respectively) and RV compared with the IVS (ratios 1.77 [1.60-1.97]; 8.41[6.35-11.13], respectively). The ratio of epicardial versus endocardial fat was increased in both ventricles (RV: ratio 4.49 [3.67-5.49]; LV: ratio 3.46 [2.49-4.81]). In multivariable analysis, there was no significant association between collagen or fat proportion and sex (p=0.12; p=0.08, respectively), age at death (p=0.36; p=0.23, respectively), or BMI (p=0.45; p=0.43, respectively).

**Conclusions:**

Our findings provide location and sex-specific proportions of myocardial histological tissue composition that may aid quantitative evaluation of pathology in future studies.

## Highlights

• Automated image analysis is a useful tool for cardiac tissue quantification.
• Collagen and fat proportions are demonstrably higher in the right ventricle.
• We present reference values for collagen and fat proportions in normal myocardium.

## 1 Introduction

Digital quantification of myocardial tissue structure has emerged as an important tool in the pathological and clinical evaluation of cardiovascular disease. Excessive accumulation of collagen within the extracellular matrix, the hallmark of myocardial fibrosis, results in pathological structural remodeling of the heart muscle. Recently, histomorphometric analysis has been employed in the quantification of tissue components and fibrosis in arrhythmogenic cardiomyopathy [1] and Brugada syndrome [2]. Semiautomated histological quantification of fibrosis has also been used to validate findings from cardiac magnetic resonance T1 mapping in patients with severe aortic stenosis [3]. To define pathology, knowledge of the relative proportion of collagen, fat, and cardiomyocytes in normal ventricular myocardium is necessary. The aim of the study was to determine histological cardiac tissue composition and collagen content using automated digital pathology software.

## 2 Material and methods

**Fig. 1:**
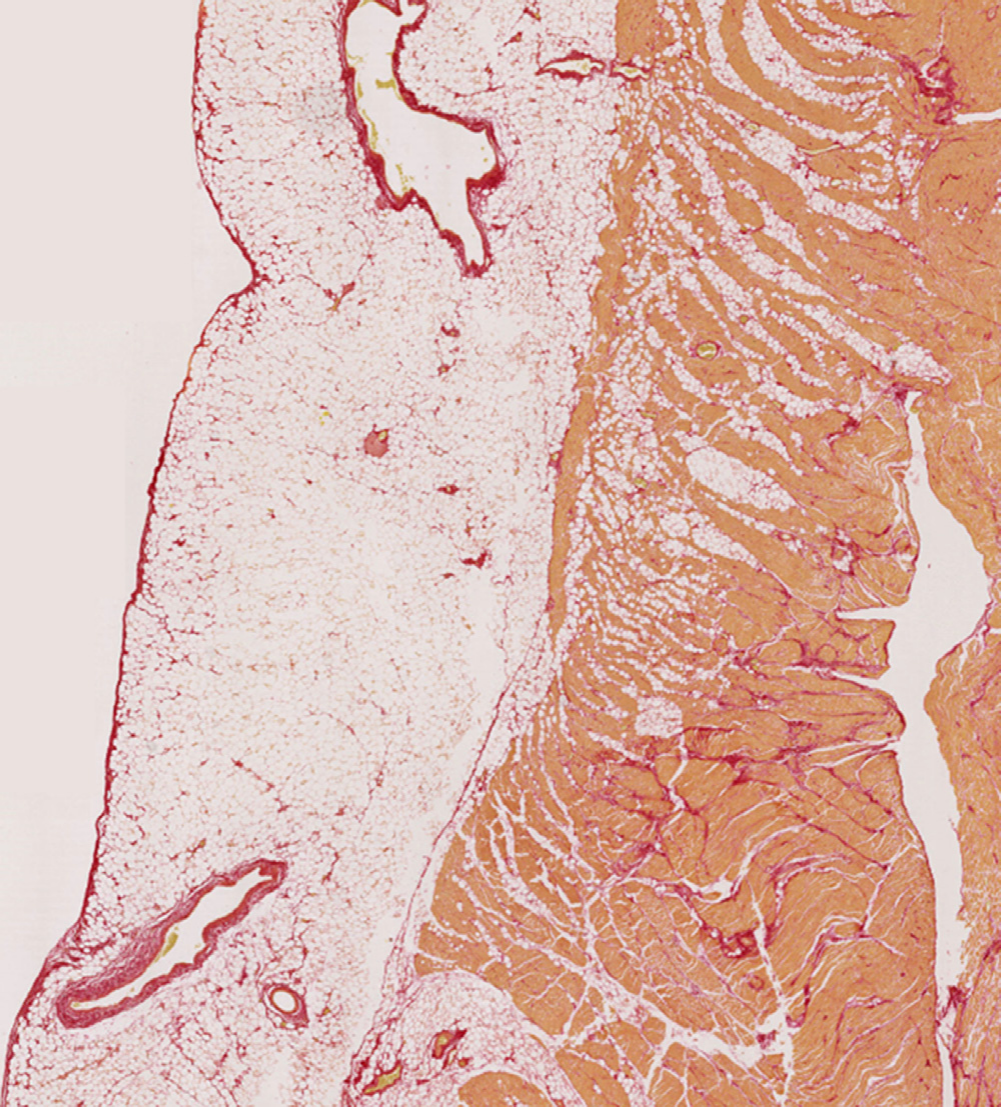
Histological slide (Picrosirius red stain) of the right ventricular free wall section from a noncardiac death decedent with a structurally normal heart at expert cardiac postmortem.

**Fig. 2:**
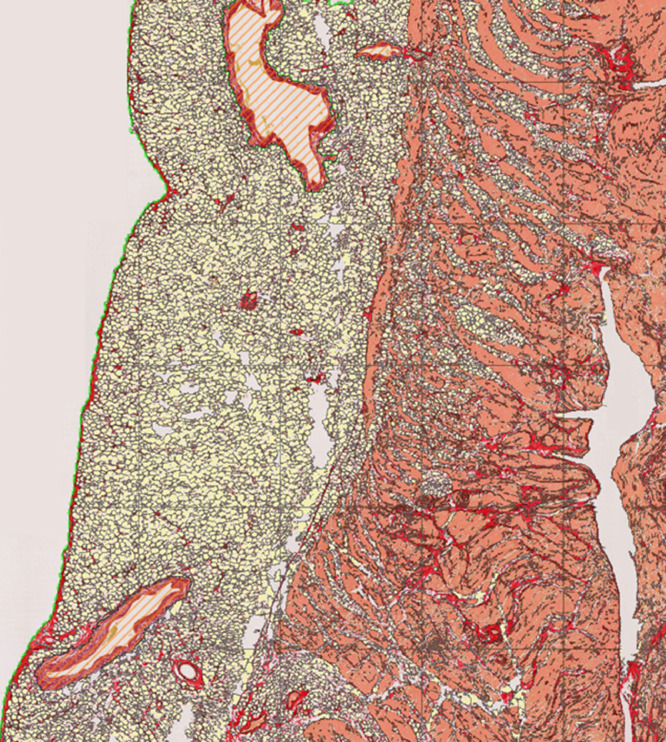
Digital characterization of the histological slide shown in Fig. 1: fat (yellow), collagen (red), and myocytes (orange). Postprocessing performed using Visiopharm software. Different color band thresholds define individual tissue components and include morphological operations for fatty tissue. Perivascular collagen (hatched orange area) was excluded based on automated detection of luminal regions (elliptical score ≥0.4) with associated red PSR stain.

We identified 29 noncardiac death cases with retained cardiac tissue referred to our national cardiac pathology center. Ethical approval for this study was obtained from the UK National Health Service Research Ethics Committee (ref. 10/H0724/38) and conformed to the principles outlined in the Declaration of Helsinki. Informed consent was provided by the next of kin at the time of referral. Demographics, clinical data, toxicological, and pathological findings were entered prospectively into a database. Whole hearts were macroscopically and microscopically normal following examination by expert cardiac pathologists. Tissue sections (5-μm thickness) were sampled at the mid-ventricular level or from the anterior aspect of the right ventricular outflow tract. In total, 6 regions were evaluated: right ventricular outflow tract; right ventricle (RV); anterior interventricular septum (IVS); posterior IVS; anterior left ventricle (LV); and posterior LV. The RV section included 3 sampled locations from the right anterior, lateral, and posterior walls. Right ventricular tissue areas were combined for the purpose of morphometric quantification. Sections were stained with Picrosirius Red (PSR) for collagen (Fig. 1) and slides were scanned using 20× magnification on an automated high-resolution scanner (Hamamatsu Nanozoomer). Automated calculation of cross-sectional tissue area and quantification of collagen (%), myocytes (%), and fat (%) was performed using an application developed within Visiopharm image analysis software (Visiopharm A/S, Hoersholm, Denmark). Perivascular collagen surrounding epicardial and intramural vessels was excluded due to high variability in vessel caliber between samples. Exclusion was based on automated lumen detection (elliptical score ≥0.4) incorporating surrounding region of PSR stain, as shown in Fig. 2. Total tissue area was categorized into epicardial and endocardial regions (50:50). Continuous data are summarized as means ± standard deviations and categorical data as groups’ percentages. Proportions (log transformed) of each tissue component were analyzed according to sampling location (LV, IVS, and RV) using a multilevel model, accounting for the clustered structure of the data. Predictions at the original scale were expressed in terms of geometric means across groups in the data.

## 3 Results and discussion

The majority of decedents were male (25/29; 86%). The mean age at death was 32.1 ± 9.9 (range 18–54) and mean BMI 28.7 ± 7.3. Most sudden deaths were due to opioid toxicity (22/29; 76%) or noncardiac trauma (3/29; 10%). The mean heart weight was 359.6 ± 67.1 g. The mean sampled tissue areas for the RV, IVS, and LV were 192.8 ± 45.2 mm^2^, 243.0 ± 80.4 mm^2^, and 240.9 ± 65.3 mm^2^, respectively. Table 1 outlines predicted values for the proportion of collagen, fat, and myocytes (total, epicardial, and endocardial) in the RV, IVS, and LV. There was insufficient evidence to support differences in collagen (*P*= .08) or fat (*P* = .06) proportion according to sex; predicted values for males (n = 25) and females (n = 4) are shown in Table 2.

**Table 1:** Predicted values (geometric means and corresponding 95% CI) of cardiac tissue composition within the right ventricle, septum, and left ventricle (n = 29 noncardiac death cases)

		Right ventricle		Septum		Left ventricle	
		Mean	95% CI	Mean	95% CI	Mean	95% CI
Collagen % of tissue area	Total	15.2	13.2–17.5	8.6	7.4–9.9	9.5	8.2–10.9
	Epicardial	15.9	13.6–18.5			9.1	7.8–10.6
	Endocardial	13.5	11.6–15.8			9.5	8.1–11.1
Fat % of tissue area	Total	12.3	9.7–15.6	1.5	1.2–1.9	4.7	3.7–5.9
	Epicardial	20.1	15.7–25.6			7.2	5.6–9.1
	Endocardial	4.5	3.5–5.7			2.1	1.6–2.6
Myocytes % of tissue area	Total	65.9	63.2–68.8	88.6	85.0–92.4	81.2	77.8–84.6
	Epicardial	52.1	48.5–55.9			74.3	69.3–79.7
	Endocardial	79.5	74.0–85.4			86.8	81.0–93.1

CI indicates confidence intervals of the predictions.

**Table 2:** Predicted values of collagen and fat proportions by sex (geometric means and corresponding 95% CI) within the right ventricle, septum, and left ventricle (n = 25 males; n = 4 females)

			Right ventricle		Septum		Left ventricle	
			Mean	95% CI	Mean	95% CI	Mean	95% CI
Males	Collagen % of tissue area	Total	14.5	12.6–16.7	8.2	7.1–9.5	9.1	7.8–10.5
		Epicardial	15.1	12.9–17.7			8.7	7.4–10.1
		Endocardial	12.9	11.0–15.1			9.1	7.8–10.6
	Fat % of tissue area	Total	11.5	9.0–14.8	1.4	1.1–1.8	4.4	3.4–5.6
		Epicardial	18.9	14.8–24.2			6.8	5.3–8.6
		Endocardial	4.2	3.3–5.4			2.0	1.5–2.5
Females	Collagen % of tissue area	Total	20.1	14.3–28.1	11.3	8.1–15.9	12.5	8.9–17.5
		Epicardial	21.1	14.8–30.2			12.1	8.5–17.3
		Endocardial	18.1	12.6–25.8			12.7	8.9–18.1
	Fat % of tissue area	Total	18.7	11.4–30.6	2.2	1.4–3.6	7.1	4.4–11.7
		Epicardial	28.5	18.2–44.7			10.2	6.5–16.0
		Endocardial	6.3	4.1–10.0			3.0	1.9–4.6

CI indicates confidence intervals of the predictions.

The proportion of collagen was higher in the RV compared with the LV (ratio 1.61; 95% confidence interval [CI] 1.45–1.78; *P*< .001), RV compared with the IVS (ratio 1.77; 95% CI 1.60–1.97; *P* < .001), and RV epicardium compared with endocardium (ratio 1.17; 95% CI 1.05–1.30; *P*= .004). There was insufficient evidence to support differences in collagen proportion between the IVS and LV (*P*= .06). The LV epicardium and endocardium contained similar proportions of collagen (*P*= .34). Fatty tissue was increased in the RV compared with the LV (ratio 2.63; 95% CI 1.99–3.48; *P* < .001) and RV compared with the IVS (ratio 8.41; 95% CI 6.35–11.13; *P*< .001). The ratio of epicardial versus endocardial fat was increased in both ventricles (RV: ratio 4.49; 95% CI 3.67–5.49; *P* < .001; LV: ratio 3.46; 95% CI 2.49–4.81; *P* < .001). In multivariable analysis, there was no significant association between collagen or fat proportion and sex (*P* = .12; *P* = .08, respectively), age at death (*P* = .36; *P* = .23, respectively), or BMI (*P* = .45; *P* = .43, respectively).

Our results demonstrated that RV collagen and fat proportions were significantly higher than the LV, particularly within the RV epicardium (15.9% and 20.1%, respectively). This is in line with earlier observations reporting higher concentrations of collagen within the right heart [4]. While stereological techniques are recognized as the gold standard in collagen quantification [5], we used a morphometric and automated approach that included analysis of whole tissue sections. Our data did not yield sex-specific differences in collagen and fat proportion, which may be due to the small number of females included in our study. This is reflected in the respective CIs shown in Table 2.

Overall, our study highlights the spectrum of normal variation of collagen and fat content within the RV, in contrast to the pathological changes seen in diseases such as arrhythmogenic cardiomyopathy [6]. Although we have previously reported lower ventricular collagen proportions in a control group of 6 structurally normal hearts [2], this analysis was performed using semiautomated color thresholds. Lower proportions may reflect the imprecise nature of semiautomated approaches to collagen quantification, which can underestimate collagen area and affect reproducibility. Indeed, a recent study reported an approximate 50% reduction of detected collagen area using a semiautomated approach when compared with automated or stereological light microscopy techniques [7]. Moreover, the effect of tissue thickness on collagen quantification should not be understated. In the aforementioned study, there was a positive relationship between automated collagen quantification and increasing section thickness. This underscores the importance of using thin sections of paraffin-embedded tissue and maintaining consistency across samples. Our data highlight the potential utility of automated histological quantification to complement expertise provided by the pathologist and should be interpreted in the context of thin tissue sections and comparable sampling location.

## 4 Conclusions

This is the first study to report regional proportions of cardiac tissue composition in normal human ventricular myocardium. Previous work has demonstrated the validity of automated methods of histological image quantification in the assessment of myocardial fibrosis [7]. Here, we provide location and sex-specific proportions that represent reference values to aid quantitative evaluation of ostensibly diseased samples in future studies.

## Author contribution

C.M., J.W., P.B., and A.J. were involved in the conception, design, and drafting of the article. C.M., A.A., M.P., S.S., E.R.B., and M.N.S. were involved in the interpretation of data and critical revision of content. I.C.S. supervised all the statistical aspects of this study including design; analyses were conducted by C.M. All authors have read and approved the final article.

## Funding

CM is funded by the British Heart Foundation (BHF Clinical Research Training Fellowship FS/18/28/33549). Cardiac Risk in the Young UK (CRY) provides funding to support the CRY Centre for Cardiac Pathology.

## Disclosures

None.
